# Handgrip Strength is an Independent Predictor of Functional Outcome in Hip-Fracture Women: A Prospective Study With 6-Month Follow-Up

**DOI:** 10.1097/MD.0000000000000542

**Published:** 2015-02-13

**Authors:** Marco Di Monaco, Carlotta Castiglioni, Elena De Toma, Luisa Gardin, Silvia Giordano, Rosa Tappero

**Affiliations:** From the Division of Physical Medicine and Rehabilitation (MDM, CC, RT); and Service of Occupational Therapy (EDT, LG, SG), Presidio Sanitario San Camillo, Turin, Italy.

## Abstract

The objective of this study was to investigate the contribution of handgrip strength in predicting the functional outcome after hip fracture in women.

We prospectively investigated white women (N = 193 of 207) who were consecutively admitted to a rehabilitation hospital after a hip fracture. We measured handgrip strength with a Jamar dynamometer (Lafayette Instrument Co, Lafayette, IN), on admission to rehabilitation. Ability to function in activities of daily living was assessed by the Barthel index both on discharge from rehabilitation and at a 6-month follow-up.

We found significant correlations between handgrip strength measured before rehabilitation and Barthel index scores assessed both on discharge from rehabilitation (ρ = 0.52, *P* < 0.001) and after 6 months (ρ = 0.49, *P* < 0.001). Significant associations between handgrip strength and Barthel index scores persisted after adjustment for age, comorbidities, pressure ulcers, medications in use, concomitant infections, body mass index, hip-fracture type, and Barthel index scores assessed both preinjury and on admission to rehabilitation (*P* = 0.001). Further adjustments for both Barthel index scores and Timed Up-and-Go test assessed at rehabilitation ending did not erase the significant association between handgrip strength and the Barthel index scores at the 6-month evaluation (*P* = 0.007). To define successful rehabilitation, we categorized the Barthel index scores as either high (85 or higher) or low (<85). The adjusted odds ratio for 1 SD increase in grip strength was 1.73 (95% confidence interval [CI] 1.05–2.84, *P* = 0.032) for having a high Barthel index score at the end of inpatient rehabilitation and 2.24 (95% CI 1.06–5.18) for having a high Barthel index score at the 6-month follow-up.

Handgrip strength assessed before rehabilitation independently predicted the functional outcome both after inpatient rehabilitation and at a 6-month follow-up in hip-fracture women.

## INTRODUCTION

Hip fracture in older people is a major concern for health care systems in several countries because it is associated with a 8% to 36% excess mortality within 1 year.^1^ Beyond mortality, hip fracture represents a key risk factor for new falls and fractures, including recurrent fractures at the hip.^2^ Furthermore, the fracture is often followed by permanent restriction in both activity and participation, and approximately 20% of the hip-fracture survivors require long-term nursing home care, whereas only 40% fully regain their preinjury level of independence.^2^

Defining the functional prognosis of hip-fracture survivors is a crucial issue to optimize musculoskeletal rehabilitation: it enables clinicians to inform patients, select treatment interventions, set rehabilitation objectives, plan a proper discharge program, and obtain early information on the needs for home adjustments and community support.^3^ Moreover, predicting the functional outcome helps to estimate the burdens for health care systems, allocate resources, and define optimal setting and care organization.^4^

A number of independent predictors have been associated with the functional outcome after hip fracture. Among them, a limited number of studies have pointed out low muscle strength as a negative prognostic factor.^5–10^ Other variables significantly associated with unfavorable function include older age, reduced prefracture level of functional autonomy, cognitive impairment, postoperative pain, incident falls and fear of falling, poor nutritional state, vitamin D depletion, depression, postoperative delirium, poor social support, prevalent vertebral fractures, a long hospital stay, male sex, and several comorbidities.^11–15^

Muscle strength can be assessed by various techniques at different body sites. Although lower limbs are more relevant than upper limbs for gait and physical function, handgrip strength is measured very easily, and it has been widely used as a reliable and feasible surrogate for whole body strength.^16,17^ Indeed, isometric handgrip strength is strongly related with lower extremity muscle power, knee extension torque, and calf cross-sectional muscle area.^18^

Our aim was to investigate the independent contribution of handgrip strength in predicting the functional outcome after hip fracture in women. We hypothesized that handgrip strength assessed at admission to inpatient rehabilitation could predict the ability to function in activities of daily living both at the end of inpatient rehabilitation and at a 6-month follow-up independently of several confounders.

## METHODS

### Patients

The study was performed in a city with about 1 million inhabitants. We evaluated 207 white women without cognitive impairment (Mini Mental State Examination Test score >23) and without prevalent motor impairment due to neurologic diseases, consecutively admitted to our physical medicine and rehabilitation division because of a hip fracture during a 12-month period (between January and December 2013). We focused on white patients because few nonwhite elderly subjects live in Italy. The women came from several orthopedic wards from various hospitals, and were referred for acute inpatient rehabilitation by the consultant physiatrists of the orthopedic wards. The criteria agreed upon for selecting hip-fracture women to undergo acute inpatient rehabilitation were health conditions allowing a total of 3 hours of physical therapy and/or occupational therapy daily, weight-bearing to tolerance on the fractured hip, and a potential high increase in the ability to function in activities of daily living due to an intensive rehabilitation regimen.

Ten of the 207 women we evaluated were excluded from our study because their hip fractures resulted from either major trauma or cancer affecting bone. The remaining 197 women sustained fractures that either were spontaneous or resulted from minimal trauma (trauma equal to or less than a fall from a standing position). They all gave their informed consent to participate in the study. Four women could not complete inpatient rehabilitation because of acute concomitant diseases. The final study sample included 193 women whose data were included in the main analyses.

Our rehabilitation protocol included 3 hours a day for 5 days a week of physical exercise to improve strength and balance, advice and training on the use of assistive devices, and training in mobility tasks and activities of daily living conducted by physical therapists and occupational therapists. At least 3 hours during the stay in the rehabilitation hospital were dedicated by a skilled occupational therapist to suggest targeted modifications of home environment and behavioral changes to prevent falls. The criterion for discharge from rehabilitation was the achievement of the highest possible Barthel index score (as judged by the responsible physiatrist) in the 3 following items: dressing, transfers, and walking. Institutional review board (Presidio Sanitario San Camillo, Turin, Italy) approval was obtained for the study protocol.

### Outcome Measures

Handgrip strength was measured with a Jamar hand dynamometer within the third day since admission to the rehabilitation hospital. Testing was performed with the participant in sitting position and with her shoulder adducted and neutrally rotated, elbow flexed at 90° with the forearm in neutral position, and wrists between 0° and 30° of flexion and between 0° and 15° of ulnar deviation. The best recorded of 3 attempts of maximal voluntary contraction, performed at 1-minute intervals at nondominant arm, was considered for analyses.

In each patient, we recorded age, number of medications in use, presence of pressure ulcers (stage 2 or higher according to the classification from the National Pressure Ulcer Advisory Panel), number of concomitant diseases (all the prevalent diseases judged clinically relevant during the length of stay), infections (at least 1 infection needing antibiotic therapy during the stay in hospital), body mass index, and hip-fracture type (either cervical or trochanteric on the basis of both radiologic and surgical findings).

Functional evaluation, at both rehabilitation admission and discharge from the rehabilitation hospital, was assessed by skilled physiatrists by using the Barthel index (original version unchanged). Also, the physiatrists assessed preinjury Barthel index scores by anamnesis. At a 6-month follow-up, the Barthel index scores were reevaluated by a skilled occupational therapist by telephone interviews in 148 of the 193 women. Data from 45 women were not available at the 6-month follow-up because they were not found (N = 25), had acute severe concomitant diseases (N = 6), died during the 6-month period (N = 4), or refused to participate in the telephonic interview (N = 10). The Barthel index assesses basic activities of daily living; its score ranges from 0 (total dependence) to 100 (total independence). The assessors were not aware of the results of handgrip strength measure at the time of Barthel index score evaluation.

A Timed Up-and-Go (TUG) test was performed at the end of the rehabilitation course to determine the amount of time required for the subject to rise from a standard armchair, walk 3 meters away, turn, return, and sit down again. One practice and 1 test trial were performed for each participant. Before testing, a trained evaluator provided standardized verbal instructions regarding the test procedures. Time was recorded with a stopwatch started on the command “ready-set-go” and stopped as the participant sat down. Data were available for 167 of the 193 women. The remaining 26 women could not perform the TUG test because they were not able to walk without assistance.

### Data Analysis

We assessed the linear correlation between handgrip strength and Barthel index scores, by using a Spearman rank test, taking into account the nonnormal distribution of the outcome variables in our sample as shown by a Shapiro–Wilk test. Additionally, handgrip strength was included in a linear multiple regression model as an independent variable together with 9 potential confounders: Barthel index scores assessed both preinjury and on admission to rehabilitation, age, number of medications in use, presence of pressure ulcers, number of comorbidities, presence of concomitant infections, body mass index, and hip-fracture type. The dependent variable in the regression model was the Barthel index score on discharge. Because the Barthel index score was nonnormally distributed, area transformation was performed, using the formula (r-1/2)/w, where w is the number of observations and r is the rank.^6,13^ Linear multiple regression was also performed after substituting the Barthel index scores assessed at the 6-month follow-up (after normalization by area transformation) for the Barthel index score assessed at the end of inpatient rehabilitation. A further regression model with the Barthel index scores at the 6-month follow-up as the dependent variable included 2 additional covariates: Barthel index scores and TUG test both assessed on discharge from rehabilitation. Following area transformation of the dependent variables, the residuals were normally distributed in the regression models. Homoscedasticity was verified by plotting the residuals against the predicted values: the variance of the residuals looked homogeneous across levels of the predicted values. Collinearity diagnostics showed that the percent of variance in each predictor that could not be accounted for by the other predictors was always >75% (no redundant predictors were found). We had no missing data.

A further analysis was performed after categorization of the Barthel index scores into 2 groups both at the end of inpatient rehabilitation and at the 6-month follow-up: lower than 85 (“low score”) or 85 or higher (“high score”). The association between handgrip strength and high or low Barthel index score was adjusted for the 9 confounders listed above using a binary logistic regression because the dependent variable was dichotomous (having either a low or a high score). Handgrip strength expressed in SD units was included into the binary logistic regression model as an independent variable together with the 9 potential confounders.

The statistical package used was SPSS, version 14 (SPSS Inc, Chicago, IL).

## RESULTS

Descriptives for the 193 women are shown in Table 1.

**TABLE 1:**
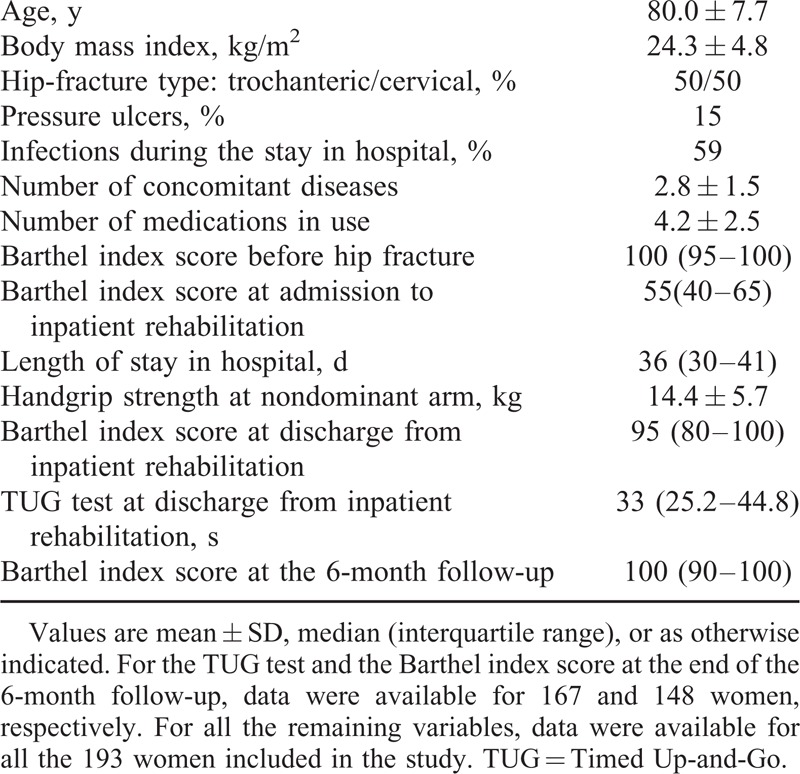
Characteristics of the Women Included in the Study

We found a significant positive correlation between handgrip strength measured at the nondominant arm on admission to rehabilitation and the Barthel index scores assessed both on discharge from rehabilitation (ρ = 0.52, *P* < 0.001) and at the 6-month follow-up (ρ = 0.49, *P* < 0.001). The significant association between handgrip strength and the functional score persisted after adjustments for 9 potential confounders (Tables 2 and 3). Further inclusion of both Barthel index scores and TUG test assessed at the end of inpatient rehabilitation did not erase the significant association between handgrip strength and the Barthel index scores assessed 6 months after hospital discharge (Table 4).

**TABLE 2:**
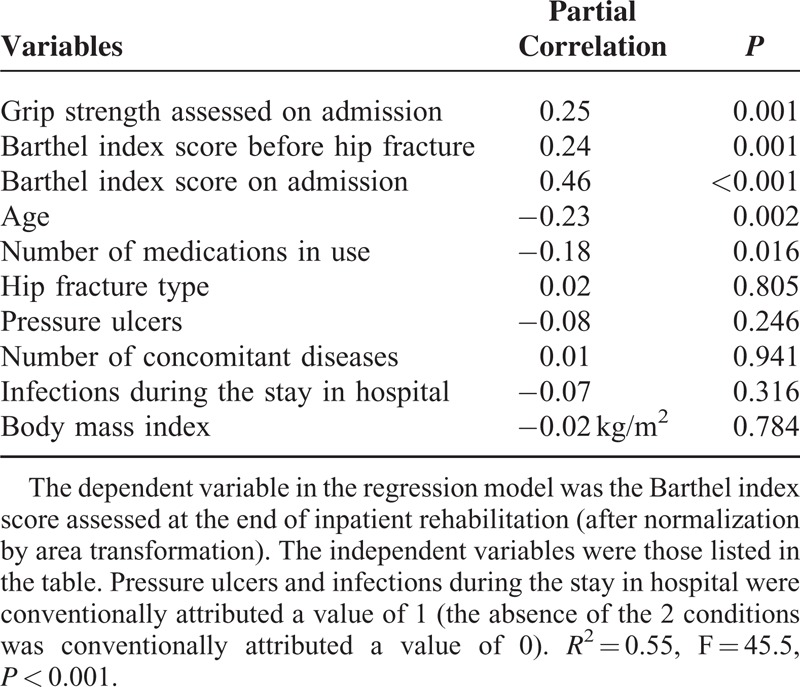
Association Between Handgrip Strength Measured Before Rehabilitation and Barthel Index Scores Assessed at the End of Inpatient Rehabilitation

**TABLE 3:**
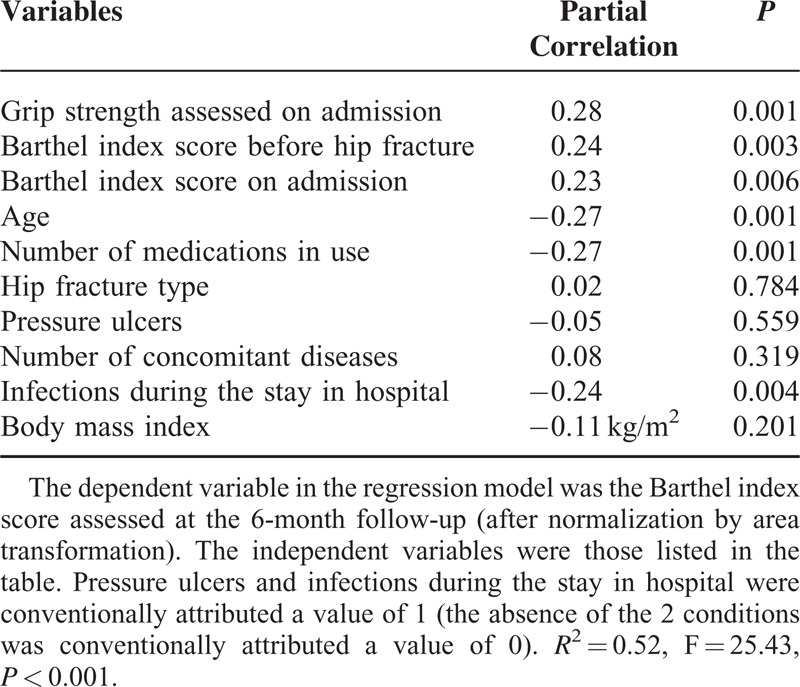
Association Between Handgrip Strength Measured Before Rehabilitation and Barthel Index Scores Assessed at the 6-Month Follow-Up

**TABLE 4:**
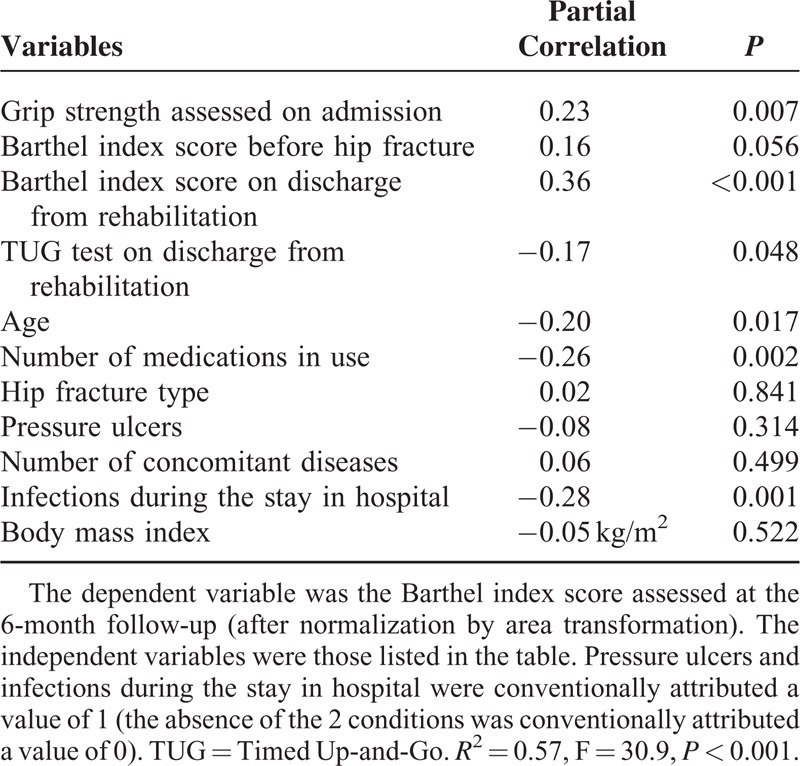
Association Between Handgrip Strength Measured Before Rehabilitation and Barthel Index Scores Assessed at the 6-Month Follow-Up With Further Adjustments

Handgrip strength assessed on admission to rehabilitation was significantly associated with the Barthel index scores categorized as either high (85 or higher) or low (<85). After adjustment for 9 potential confounders, the odds ratio for 1 SD increase in grip strength (5.7 kg) was 1.73 (95% confidence interval [CI] 1.05–2.84, *P* = 0.032) for having a high Barthel index score at the end of inpatient rehabilitation (Table 5) and 2.24 (95% CI 1.06–5.18) for having a high Barthel index score at the 6-month follow-up (Table 6).

**TABLE 5:**
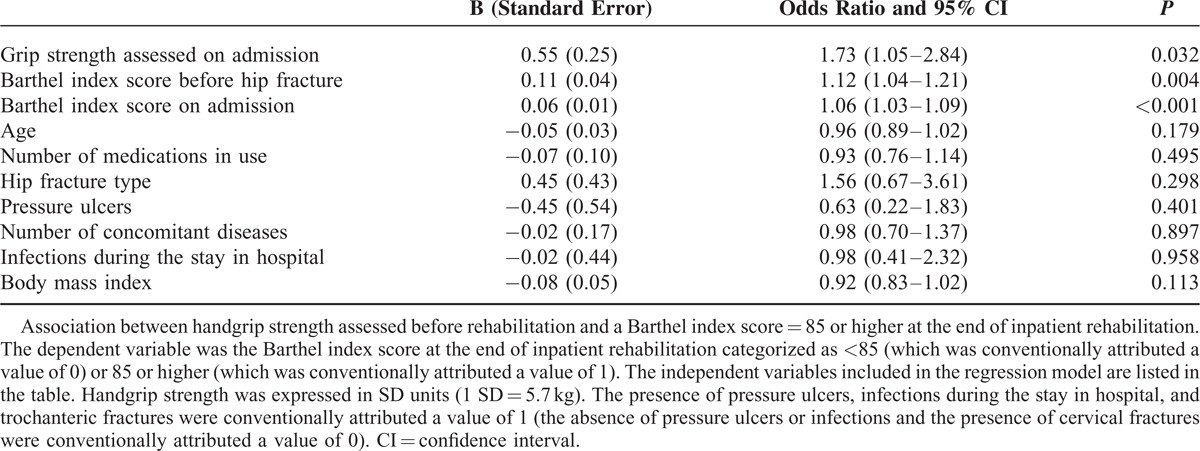
Binary Logistic Regression Analysis

**TABLE 6:**
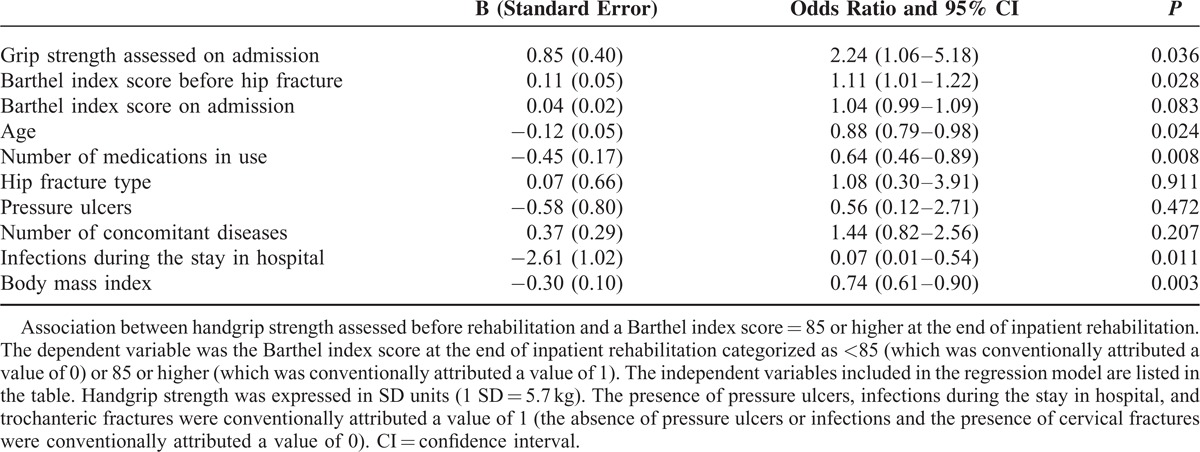
Binary Logistic Regression Analysis

## DISCUSSION

Data show that handgrip strength measured before rehabilitation was significantly associated with the ability to function in activities of daily living assessed both at the end of inpatient rehabilitation and at a 6-month follow-up in hip-fracture women.

The prognostic role we describe is in agreement with 4 previous reports that assessed isometric grip strength after hip-fracture surgery and various measures of functional outcome at different time points. Wehren et al^8^ found that handgrip strength predicted the self-reported ability to function in activities of daily living during a 12-month follow-up in 205 women. Savino et al^10^ showed that handgrip strength significantly predicted walking recovery at a 1-year follow-up in 504 patients. Beloosesky et al^9^ showed that handgrip strength was significantly associated with the functional independence measure score assessed 6 months later in 105 women. Di Monaco et al^6^ reported a significant association between grip strength and the Barthel index scores at the end of acute inpatient rehabilitation in 123 women.

The originality of our data rests on the adjustments we performed. We included grip strength as an independent variable in regression models together with 9 potential confounders, and we show that the prognostic role of grip strength was independent of the covariates. At a step further, we added both Barthel index scores and TUG test assessed after inpatient rehabilitation to the panel of functional predictors, and handgrip strength assessed before rehabilitation kept its independent predictive role. This is noteworthy because inpatient rehabilitation accounts for the majority of the changes in functional abilities after hip fracture,^19^ but we show that neither functional autonomy nor lower-limb performance measured after rehabilitation erased the prognostic role of grip strength assessed before rehabilitation.

The clinical meaning of our study regards 2 aspects. First, we emphasize the relevancy of measuring handgrip strength to estimate the functional prognosis after hip fracture: handgrip strength is easily assessable and inexpensive,^18^ and it emerges as an independent predictor of function. The odds of achieving a “high” functional score (Barthel index score = 85 or higher) roughly doubled for each SD increase in grip strength. Although there is not a threshold for Barthel index scores universally agreed upon, several authors have indicated a cut point of 85 to define successful rehabilitation.^13,20–22^ Notably, the effect of handgrip strength was shown after multiple adjustments. Overall, the panel of prognostic factors we included in the regression model predicted 57% of the variance in Barthel index scores at the 6-month follow-up. Future prospective studies should aim at increasing the percentage of the variance in validated measures of functional outcome predicted by an early-stage examination. Likely, grip strength should be considered in any future predictive models.

The second clinical meaning regards the rationale for interventions aimed at increasing muscle strength to optimize the functional recovery after hip fracture, given the link between muscle weakness and unfavorable outcome. In line with this observation, Visser et al^7^ showed that grip-strength changes paralleled the changes of a self-reported 5-item mobility score during a 12-month period in 90 women following a hip fracture. Absolute values of handgrip strength were low in our sample,^16^ as expected in hip-fracture patients who are usually frail.^23^ Strengthening exercises performed either alone^24–28^ or as a part of multidimensional interventions^29,30^ actually had favorable effects on several outcome measures after hip fracture. This is in agreement with several reports in other frail subjects: strengthening exercises effectively counteracted age-related muscle loss improving both strength and performance, with modest requirements of time (sessions of 30 minutes twice per week) and equipment.^17,31,32^

Among the covariates we included in our regression models, a significant prognostic role was found for Barthel index scores before fracture occurrence, at the beginning or at the end of inpatient rehabilitation, age, and 2 markers of comorbidity (number of medications in use and infections during the stay in hospital). These findings are in agreement with several previous reports.^11^

Our study has limitations. We evaluated white, cognitively intact women without neurologic impairment admitted to a single rehabilitation hospital in Italy, who were surgically operated on, and who were referred for inpatient rehabilitation. As a consequence, our data cannot be generalized to the overall population of patients who sustain hip fractures. We adjusted our results for several prognostic factors, but we did not collect data on other potential confounders, including nutritional state, prevalent vertebral fractures, depression, pain, social support, fear of falling, and balance confidence.^11^ We assessed the functional recovery after hip fracture by using only 1 functional scale (ie, the Barthel index). However, the Barthel index is a validated scale in hip-fracture patients,^33^ and it has been widely used to evaluate functional outcome, the role played by several prognostic factors in affecting the functional outcome, and functional progress caused by either rehabilitation or other treatments after hip fracture.^11^ We did not assess any measures of participation or health-related quality of life.

In conclusion, despite limitations of our study, data support the prognostic role of grip strength at admission to inpatient rehabilitation after a hip fracture. Models aimed at predicting the functional outcome in hip-fracture survivors should consider handgrip strength, although a number of factors have been shown to play a prognostic role,^11^ and the independency of handgrip strength should be further examined. Together with other prognostic factors, grip strength may be helpful in selecting patients for proper rehabilitation protocols and settings. The unfavorable prognostic role of low muscle strength emphasizes the relevancy of resistance training to optimize recovery. Further research studies are needed to elucidate the characteristics of optimal strengthening exercise regimens.
